# In Response to “Photosensitizing Drugs and Risk of Skin Cancer in Women—A Prospective Population‐Based Study”

**DOI:** 10.1111/phpp.70021

**Published:** 2025-04-08

**Authors:** Stephen N. Birrell

**Affiliations:** ^1^ Senior Breast Cancer Research Fellow, Dame Roma Mitchell Research Laboratories University of Adelaide Adelaide Australia

Dear Editor,

I would like to thank Dr. Christensen and colleagues for their valuable contribution to the literature with their recent study examining the association between photosensitizing medications and skin cancer in women. Prospective, registry‐linked cohort studies like this are foundational for population‐level insights, and their methodological care in controlling for UV exposure and phenotypic traits is commendable.

However, I would like to highlight several critical concerns regarding the interpretation and communication of their findings on hormone replacement therapy (HRT), with specific attention to estrogen and progestogen classification, endocrine biology, and clinical impact.

Their conclusion that “estrogen treatment increased the risk of all investigated skin cancers” is based on modest hazard ratios (HRs 1.23–1.35), of which only the Basal Cell Carcinoma (BCC) association with estrogen remained statistically significant after Bonferroni correction. Yet this finding is generalized throughout the paper in a manner that may unduly alarm readers and clinicians. No absolute risk figures were provided to contextualize the practical significance of these findings. As such, this framing risks deterring appropriate and beneficial use of HRT—particularly transdermal estradiol formulations, which differ substantially from oral preparations in pharmacokinetics, metabolic effects, and potentially tissue‐specific estrogen receptor activity.

One of the most significant issues in this manuscript is the interchangeable use of the term “progesterone” when referring to all progestogenic agents, despite the well‐established biological distinctions between synthetic progestins and natural (bioidentical) progesterone. The misclassification of these compounds has profound implications for interpreting risk data and informing clinical decision‐making. This study appears to include only synthetic progestins—most likely medroxyprogesterone acetate (MPA) or norethisterone derivatives—given that natural progesterone is not readily available in Sweden. This point should be made explicitly, particularly as synthetic progestins can have anti or pro‐androgenic, glucocorticoid, or estrogenic off‐target effects that native progesterone does not share. We have proposed that the observed excess of breast malignancies associated with combined HRT may be explained, in part, by synthetic progestins such as MPA acting as endocrine disruptors to negate the protective effects of androgen signaling in the breast. Understanding the role of androgen signaling in the breast and how this is modulated by synthetic progestins is necessary to determine how combined HRT alters breast cancer risk [1]. Extrapolating these findings to skin biology, one could speculate that synthetic progestins may potentiate carcinogenic responses to UV exposure in ways that native progesterone does not. We further have supported this mechanistic divergence [2]. Demonstrating that the progesterone receptor (PR) can modulate estrogen receptor alpha (ERα) activity by redirecting ER binding to alternative genomic sites in breast cancer cells, leading to growth inhibition. This implies that progesterone is not simply a passive partner to estrogen but can reprogram estrogen's transcriptional effects—potentially counterbalancing estrogenic stimulation in epithelial tissues, including the breast and perhaps the skin. This is biologically plausible given that the skin and breast both derive from embryologic ectoderm and share extensive hormone receptor expression, including ERα, ERβ, PR, and AR (androgen receptor). Skin is not hormonally inert, and PR signaling likely plays a role in UVR response, collagen synthesis, and immune surveillance. The presence of steroid hormone receptors in skin tumors may just represent a residual remnant of cellular, physiological regulation rather than being causative of carcinogenesis, as these receptors are present in many malignancies, such as bowel carcinoma. By omitting this critical distinction and grouping synthetic progestins under the banner of “progesterone,” the study undermines translational clarity and may perpetuate misunderstanding of risk associated with bioidentical HRT formulations—especially those using transdermal estradiol with oral micronized progesterone (the formulation endorsed by many contemporary menopause societies).

The study also lacks data on oral contraceptive use, which likely affects most of the included cohort given their birth years (1925–1965). OCPs increase sex hormone binding globulin substantially, resulting in long‐term suppression of free testosterone. Given that testosterone plays a vital role in skin barrier function, sebocyte activity, and melanocyte regulation, this could confound the interaction between exogenous estrogen exposure and skin cancer risk. Not accounting for this widespread background hormonal suppression may skew results—particularly if prior OCP use predisposes the skin to estrogen‐driven photodamage due to lower androgen buffering.

## 1 Recommendations and Framing Require Caution

The conclusion that physicians should “advise their female patients prescribed estrogen…to limit sun exposure” implies causality and clinical urgency that the data do not support. Sun protection is already standard for all individuals, and it is not clear from the results that estrogen amplifies UVR risk in a clinically meaningful way independent of other variables.

## 2 A More Appropriate and Responsible Clinical Framing Would Be

Given a modest observed association between estrogen use and BCC risk, further studies are needed to determine causality, differentiate between hormonal formulations, and explore mechanisms. Standard photoprotection should be emphasized for all patients regardless of HRT status.

Given the persistent undertreatment of menopausal women due to fears—often unfounded—of HRT‐associated cancer risks, research communication in this field must be especially careful. Recent position statements by the North American Menopause Society (NAMS), British Menopause Society (BMS), and International Menopause Society (IMS) have all emphasized the safety and benefits of HRT for most healthy women under age 60 or within 10 years of menopause.

The findings, while valuable, must not be used to inadvertently reinforce outdated fears or discourage appropriate hormonal care—particularly when newer formulations are biologically distinct and demonstrably safer.

## 3 Conclusion

The study is an important contribution to a nuanced and evolving conversation about photosensitizing drugs and skin health. However, we encourage greater care in terminology, acknowledgment of hormonal complexity, and attention to formulation‐specific risks. Distinguishing between estrogen types, synthetic vs. bioidentical progestogens, and considering reproductive and OCP history will be essential for generating findings that truly inform and empower clinical decision‐making.

Sincerely,

Stephen N. Birrell, MD, PhD

Senior Breast Cancer Research Fellow

Dame Roma Mitchell Research Laboratories

University of Adelaide

## Conflicts of Interest

The author declares no conflicts of interest.

## Data Availability

Data sharing not applicable to this article as no datasets were generated or analysed during the current study.
